# Life expectancy ranking of Canadians among the populations in selected OECD countries and its disparities among British Columbians

**DOI:** 10.1186/s13690-015-0065-0

**Published:** 2015-04-03

**Authors:** Li Rita Zhang, Drona Rasali

**Affiliations:** Population and Public Health Program, Provincial Health Services Authority, 700-1380 Burrard Street, Vancouver, BC V6Z 2H3 Canada; Faculty of Kinesiology and Health Studies, University of Regina, Regina, Saskatchewan Canada

**Keywords:** Life expectancy, Longitudinal comparison, Socio-economic disparity

## Abstract

**Background:**

Canada is among the world’s leading nations with the longest life expectancy at birth (LE_0_), and British Columbia (BC) ranks top among Canadian provinces and territories for LE_0_ in both men and women. This paper examined recent data as well as projected trends in LE_0_ of Canadian men and women and explored the geographic and socioeconomic disparities in LE_0_ specific to BC.

**Methods:**

Using retrospective data on LE_0_ and age-standardized mortality rates, Canada was compared to 11 other Organization for Economic Cooperation and Development (OECD) countries with the longest LE_0_. Projections were made using linear regression modelling to the year of 2023. The association between regional LE_0_ and regional socioeconomic status (SES) was examined for the province of BC using its Local Health Area (LHA) level data on SES and LE_0_.

**Results:**

In 2009, Canadian men (LE_0_: 78.7 years) and women (LE_0_: 83.3 years) ranked 7^th^ and 8^th^, respectively among the 12 OECD nations under comparison. Significantly smaller annual gains in LE_0_ contributed to the losing of their top ranks in LE_0_ for Canadian men and women in recent years, which was projected to sustain. Higher mortality risks, particularly for lung cancer and external causes of mortality among women was found for Canada compared to leading countries on these measures. Geographic variations were evident in LE_0_ in BC, and there was a significant gap of 3.6 years in the average LE_0_ for BC’s LHAs in the lowest SES tertile (78.6 years, 95% CI: 78.0-79.3) compared to those in the highest SES tertile (82.2 years, 95% CI: 81.6-82.8).

**Conclusions:**

Canada continues to remain one of the OECD countries with longest living population. With the highest LE_0_ in the country, British Columbia has an opportunity to address socio-economic disparities in LE_0_.

## Background

Life Expectancy (at birth or age 65) is a common health indicator used worldwide as an important measure in public health that captures quantitatively the survival aspect of the state of general population health. It also highlights both the progress and the gaps in total social and societal health [1]. In particular, life expectancy at birth (LE_0_) is the estimated number of years a newborn can be expected to live given the prevailing mortality rates in the population, and it is closely related to the biological, environmental and social determinants of health [2,3].

Canada is among the world’s leading nations with the longest LE_0_. With a population of over 4 million in 2011, the third most populous province of Canada located on the Pacific coast, British Columbia (BC), ranks top among the 13 Canadian provinces and territories in terms of LE_0_ for both men (80 years) and women (84 years) based on 2007–2009 data [4]. Despite BC’s national leading status in overall population longevity, there are observed and significant geographic disparities in LE_0_ in the province, the reduction of which has been identified as one of the overarching goals in the province’s Guiding Framework for Public Health [5]. At the national and international levels, lower income has been shown to be associated with shorter life expectancy [6-8]. And the growing realization that community- and system-level approaches that target the socioeconomic root causes of poor health may be more effective at improving the overall health of the population [9-12] supports a closer examination of LE_0_ by such factors in BC.

This paper first examined recent data as well as projected trends in LE_0_ of Canadian men and women to ascertain their positions in comparison to their counterparts in the other healthiest countries within the Organization for Economic Cooperation and Development (OECD). Cause-specific mortality rates were then examined with OECD-country comparisons to assess Canada’s position relative to the highest international standards of population health and to identify areas for improvement. The second part of the paper explored the geographic and socioeconomic disparities in LE_0_ specific to BC.

## Methods

### Data source

The year 2008 was the most recent year for which data are available for all OECD countries. Using data from 2008, Japan, Switzerland, Italy, Australia, Spain, France, Sweden, Canada, Norway, Austria, the Netherlands, and New Zealand ranked among the top in terms of overall population LE_0,_ and these countries all had a population size exceeding 1,000,000. They were therefore considered as peer countries for comparisons in this study. Since the estimates of LE_0_ are sensitive to population size, those OECD countries with populations less than 1 million were excluded from the list of comparison countries in this study. Annual national LE_0_ and mortality data from all available years since 1985 for these 12 selected countries were obtained from OECD.Stat 2012 [13] for longitudinal comparisons. All mortality data were expressed as age-standardized mortality rates (ASMR) to the 1980 OECD population in order to ensure comparability.

**Figure 1:**
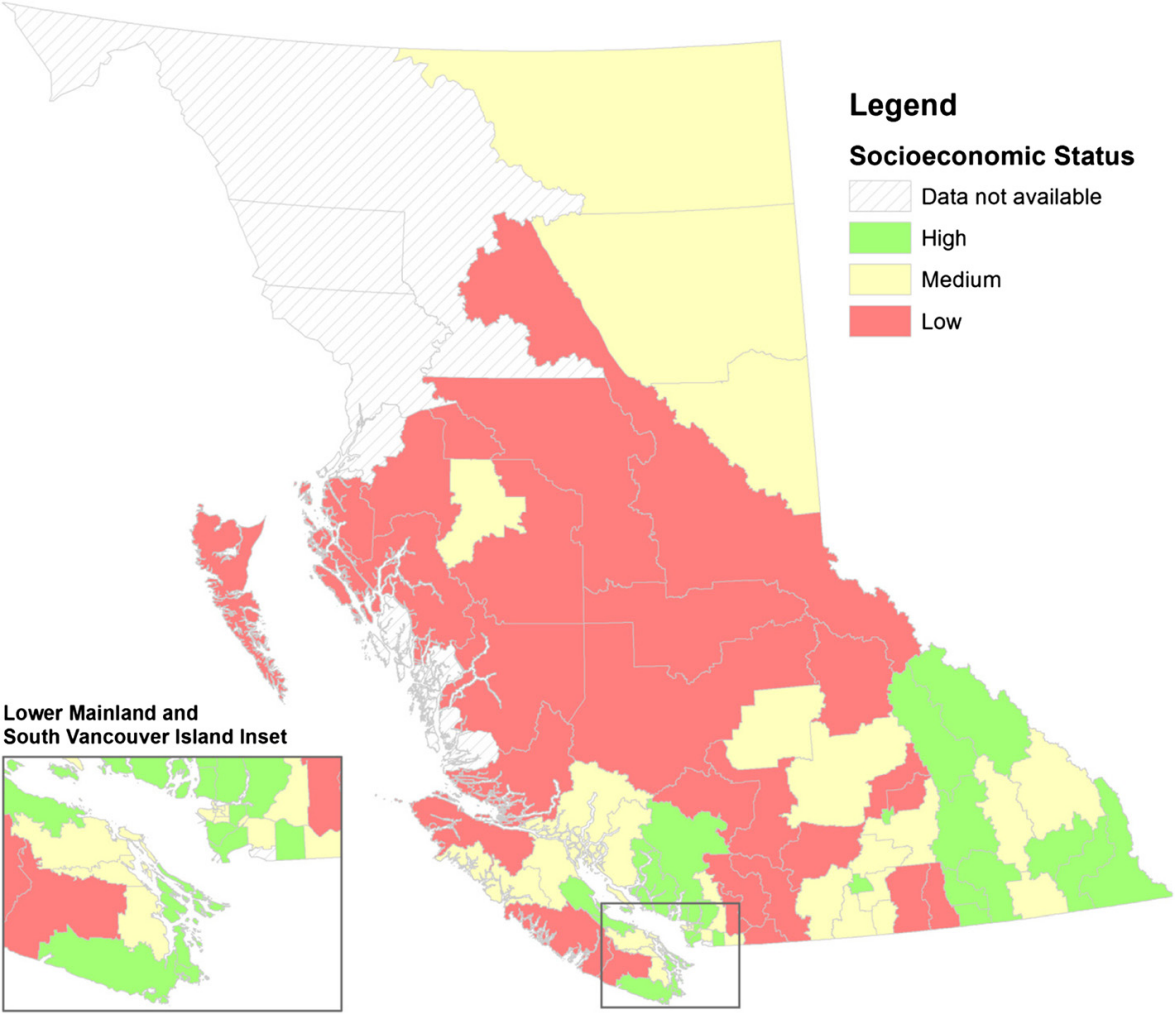
**Socioeconomic status in British Columbia by local health area, 2011.**

Sex-specific LE_0_ data averaged over 2007–2011 for the 89 local health areas (LHAs) in BC were obtained from BC Stats [14]. The overall socio-economic status (SES) index score for each LHA was obtained from BC Stats [15] that developed the index as a weighted summary of six individual indices including four basic indicators of regional hardship (human economic hardship, crime, health problems, and education concerns) and two additional indicators that highlight the target groups of children and youth. Detailed methodology for the development of the overall SES index has been published previously [15]. Since the overall SES index score was available for the whole city of Vancouver, this aggregate value was applied to all six LHAs within the city (including City Centre, Downtown Eastside, Northeast, West side, Midtown, and South Vancouver). Also, the overall SES index scores were not available for six other LHAs from elsewhere in the province (four of which are located in the north-western remote part of the province) due to their small population sizes- Snow Country, Central Coast, Stikine, Nisga’a, Telegraph Creek, and South Surrey/White Rock (please refer to Figure 1 for the locations of the LHAs with missing SES index data).

### Statistical analysis

Recent trends in LE_0_ for Canada and the other 11 OECD countries under comparison were examined from 1985 to the latest data point available (2009 for Canada, and 2010 for the others). Projections were made using simple linear regression modelling to the year 2023.

The regression slopes measuring annual improvements in LE_0_ for the regression period were used to compare the sustainability in LE_0_ gains in the different countries. To address potential multiple comparisons, analysis of covariance (ANCOVA) with year-by-country interaction was used to test statistical differences between regression slopes. Overall differences among the 12 regression slopes were confirmed first before pair-wise difference in the slope for Canada and that for each of the other 11 countries were tested at Bonferroni-adjusted significance level of 0.0045.

For all causes of mortality and each of the major cause of mortality categories as well as specific conditions within the selected categories, the difference in ASMR values between Canada and the best performing among the 12 selected countries was examined for males and females separately between 1985 and 2009.

To explore the socioeconomic disparities in LE_0_ in the province of BC, 83 LHAs with non-missing SES were categorized into 3 SES groups (low, medium, and high) using tertiles in the overall SES index scores. The overall and sex-specific average LE_0_ for the LHAs within each SES tertile were calculated and statistical differences were assessed using analysis of variance (ANOVA). Three pair-wise comparisons were made using t-test at the conservative significance level of 1%. In the sex-specific analyses for males, six additional LHAs were excluded due to the lack of LE_0_ data (Arrow Lakes, Kettle Valley, Keremeos, Princeton, Lillooet, and Queen Charlotte), and for females 12 additional LHAs were excluded due to the lack of LE_0_ data (Windermere, Kootenay Lake, Arrow Lakes, Kettle Valley, South Okanagan, Keremeos, Revelstoke, North Thompson, Queen Charlotte, Summerland, Enderby, and Fort Nelson).

The study was based on secondary analysis of publically available data, and all statistical tests were conducted using SAS 9.2 [16].

## Results

**Figure 2:**
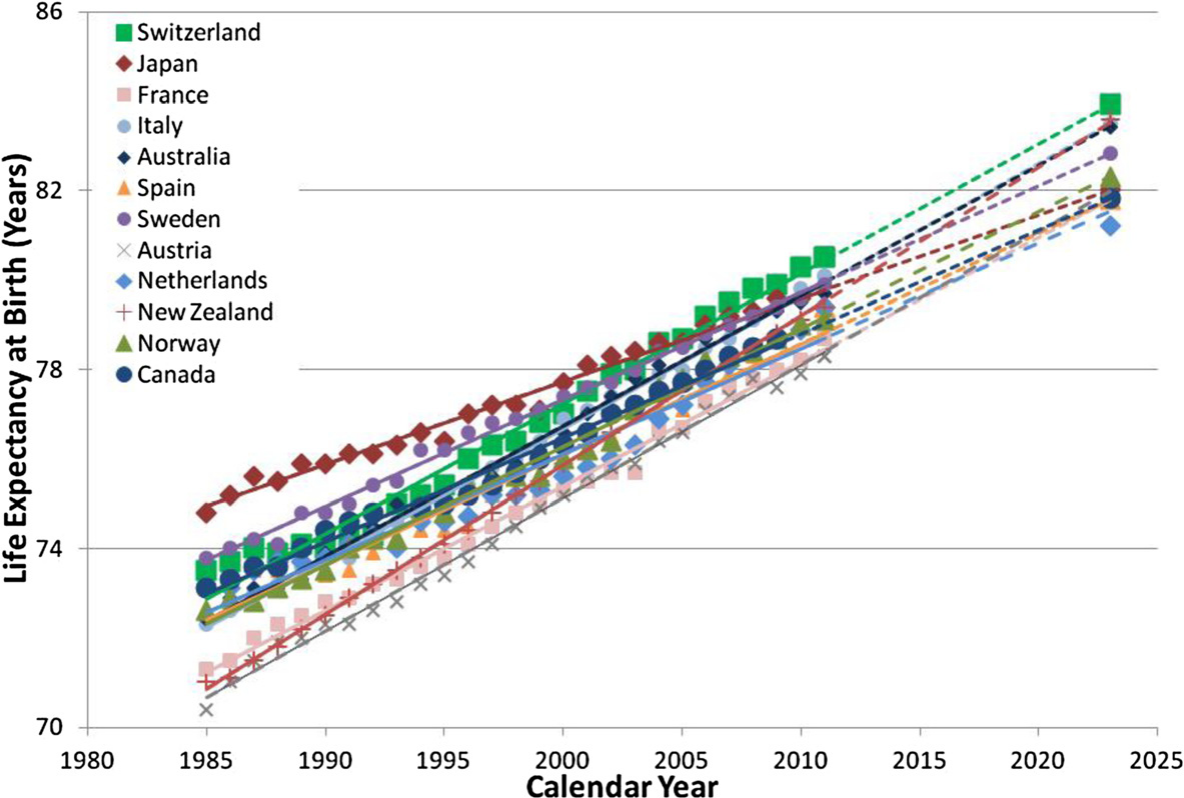
**Life expectancy at birth for men for Canada and selected healthiest countries, 1985–2023.** (solid lines show trends based on actual data, and dotted lines show trends based on projection).

**Figure 3:**
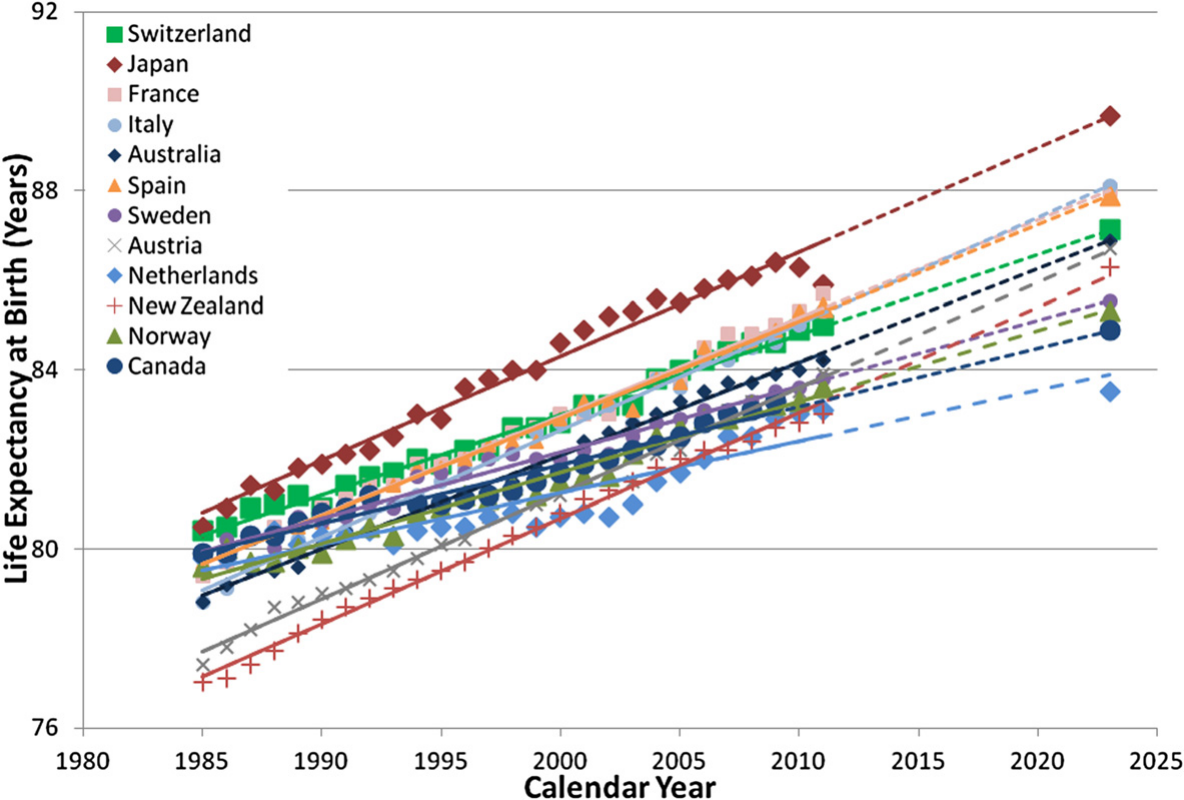
**Life expectancy at birth for women for Canada and selected healthiest countries, 1985–2023.** (solid lines show trends based on actual data, and dotted lines show trends based on projection).

Canada is among the healthiest nations in the world as measured by LE_0_ (Table 1, Figures 2 and 3)_._ In 2009, Canadian men (LE_0_: 78.7) and women (LE_0_: 83.3) ranked 7^th^ and 8^th^, respectively, among the 12 selected healthiest countries in the world. Despite the leading status on the global scale, Canadian men and women had both been losing their relatively higher ranks in LE_0_ among the peer countries under comparison in recent years. For Canadian men in particular, LE_0_ rank in comparison to the 11 other healthiest nations peaked in the early 1990’s at 3^rd^ but had been declining since, reaching the 7^th^ place in 2009.

**Table 1:** **Life expectancy at birth for Canada and selected healthiest countries, 1985–2023 estimates**

**Males**																							
	**1985**	**1986**	**1987**	**1988**	**1989**	**1990**	**1991**	**1992**	**1993**	**1994**	**1995**	**1996**	**...**	**2004**	**2005**	**2006**	**2007**	**2008**	**2009**	**R** ^**2†**^	**2023 Est.**	**(95% PI)**	
**Canada**	**73.1**	**73.3**	**73.6**	**73.6**	**74.0**	**74.4**	**74.6**	**74.8**	**n/a**	**74.9**	**75.0**	**75.2**	**...**	**77.5**	**77.7**	**78.0**	**78.3**	**78.5**	**78.7**	**99.4%**	**81.8**	**(81.5**	**- 82.2)**
**Rank** ^**¥**^	**4**	**5**	**4**	**5**	**4**	**3**	**3**	**3**	**n/a**	**5**	**4**	**6**	**...**	**7**	**7**	**7**	**6**	**6**	**7**	**n/a**	**9**		**n/a**
Australia	72.4	72.9	73.1	73.1	73.3	73.9	74.4	74.5	75.0	75.0	75.0	75.2	...	78.1	78.5	78.7	79.0	79.2	79.3	99.5%	83.6	(83.2	**-** 83.9)
Austria	70.4	71.0	71.5	71.9	72.0	72.3	72.3	72.6	72.8	73.2	73.4	73.7	...	76.4	76.6	77.1	77.4	77.8	77.6	99.7%	82.1	(81.7	**-** 82.4)
France	71.3	71.5	72.0	72.3	72.5	72.8	72.9	73.2	73.3	73.6	73.8	74.1	...	76.7	76.7	77.3	77.6	77.8	78.0	99.6%	81.7	(81.4	**-** 82.1)
Italy	72.3	72.6	73.0	73.2	73.6	73.8	73.8	74.2	74.6	74.8	75.0	75.4	...	77.9	78.0	78.5	78.7	79.1	79.4	99.8%	83.4	(83.1	**-** 83.7)
Japan	74.8	75.2	75.6	75.5	75.9	75.9	76.1	76.1	76.3	76.6	76.4	77.0	...	78.6	78.6	79.0	79.2	79.3	79.6	99.2%	82.1	(81.8	**-** 82.4)
Netherlands	73.1	73.1	73.5	73.7	73.7	73.8	74.1	74.3	74.0	74.6	74.6	74.7	...	76.9	77.2	77.7	78.1	78.4	78.7	97.5%	81.2	(80.5	**-** 81.8)
New Zealand	71.0	71.1	71.5	71.8	72.2	72.5	72.9	73.2	73.5	73.8	74.1	74.4	...	77.3	77.7	78.0	78.2	78.4	78.8	99.9%	83.6	(83.5	**-** 83.8)
Norway	72.6	72.9	72.8	73.1	73.3	73.5	74.0	74.2	74.2	74.9	74.8	75.4	...	77.6	77.8	78.2	78.3	78.4	78.7	99.3%	82.3	(81.9	**-** 82.7)
Spain	73.1	73.4	73.5	73.5	73.4	73.4	73.5	73.9	74.1	74.4	74.4	74.5	...	77.1	77.1	77.9	77.9	78.3	78.7	97.7%	81.5	(80.8	**-** 82.1)
Sweden	73.8	74.0	74.2	74.1	74.8	74.8	75.0	75.4	75.5	76.2	76.2	76.6	...	78.4	78.5	78.8	79.0	79.2	79.4	99.7%	82.9	(82.6	**-** 83.1)
Switzerland	73.5	73.7	74.0	73.9	74.1	74.0	74.2	74.3	75.0	75.2	75.4	76.0	...	78.6	78.7	79.2	79.5	79.8	79.9	99.0%	83.9	(83.3	**-** 84.4)
**Females**																							
**Canada**	**79.9**	**79.9**	**80.3**	**80.3**	**80.6**	**80.8**	**80.9**	**81.2**	**n/a**	**81.0**	**81.0**	**81.1**	**...**	**82.3**	**82.5**	**82.8**	**83.0**	**83.1**	**83.3**	**98.0%**	**84.9**	(**84.5**	**- 85.2**)
**Rank** ^**¥**^	**3**	**5**	**3**	**5**	**4**	**4**	**4**	**5**	**n/a**	**7**	**7**	**8**	**...**	**9**	**9**	**9**	**9**	**10**	**8**	**n/a**	**11**		**n/a**
Australia	78.8	79.2	79.5	79.5	79.6	80.1	80.4	80.4	80.9	80.9	80.8	81.1	...	83.0	83.3	83.5	83.7	83.7	83.9	99.6%	87.0	(86.7	**-** 87.2)
Austria	77.4	77.8	78.2	78.7	78.8	79.0	79.1	79.3	79.5	79.8	80.1	80.2	...	82.1	82.2	82.8	83.1	83.3	83.2	99.5%	86.7	(86.4	**-** 87.0)
France	79.4	79.7	80.3	80.5	80.6	80.9	81.1	81.4	81.4	81.9	81.9	82.0	...	83.8	83.8	84.5	84.8	84.8	85.0	98.7%	87.9	(87.4	**-** 88.3)
Italy	78.8	79.1	79.6	79.7	80.2	80.3	80.4	80.8	81.0	81.2	81.5	81.8	...	83.8	83.6	84.2	84.2	84.5	84.6	99.5%	88.1	(87.8	**-** 88.4)
Japan	80.5	80.9	81.4	81.3	81.8	81.9	82.1	82.2	82.5	83.0	82.9	83.6	...	85.6	85.5	85.8	86.0	86.1	86.4	99.5%	90.1	(89.8	**-** 90.4)
Netherlands	79.8	79.8	80.3	80.4	80.1	80.3	80.3	80.4	80.1	80.4	80.5	80.5	...	81.5	81.7	82.0	82.5	82.5	82.9	89.2%	83.5	(82.9	**-** 84.2)
New Zealand	77.0	77.1	77.4	77.7	78.1	78.4	78.7	78.9	79.1	79.3	79.5	79.7	...	81.8	82.0	82.2	82.2	82.4	82.7	99.8%	86.3	(86.1	**-** 86.5)
Norway	79.6	80.0	79.7	79.7	80.0	79.9	80.2	80.5	80.3	80.8	80.9	81.2	...	82.5	82.7	82.9	82.9	83.2	83.2	98.3%	85.3	(84.9	**-** 85.7)
Spain	79.7	79.9	80.3	80.3	80.5	80.6	80.8	81.3	81.4	81.8	81.8	82.0	...	83.8	83.7	84.5	84.4	84.6	84.9	99.5%	87.8	(87.5	**-** 88.1)
Sweden	79.8	80.2	80.3	80.0	80.7	80.6	80.7	81.0	80.9	81.6	81.7	81.7	...	82.8	82.9	83.1	83.1	83.3	83.5	98.6%	85.5	(85.2	**-** 85.9)
Switzerland	80.4	80.5	80.9	81.0	81.2	80.9	81.4	81.6	81.7	82.0	81.9	82.2	...	83.8	84.0	84.2	84.4	84.6	84.6	99.3%	87.1	(86.8	**-** 87.4)

^†^Represents the degree of linearity of the linear regression line.

Est. Projection estimate.

PI Prediction interval of projected LE_0_ for 2023.

^¥^Ranks are accurate to the first decimal place and ties are permitted.

The trend in LE_0_ for all countries considered in this analysis demonstrated a strong association with time measured in years (R^2^>89%) in the linear regression models. Country-specific projection to 2023 based on their recent linear trends in LE_0_ suggested that Canadian men would rank only 9^th^ among countries under comparison with a LE_0_ of 81.8 years [95% prediction interval (PI): 81.5-82.2], significantly lower than that predicted for men in Switzerland (LE_0_: 83.9, 95% PI: 83.3-84.4), New Zealand (LE_0_: 83.6, 95% PI: 83.5-83.8), Australia (LE_0_: 83.6, 95% PI: 83.2-83.9), Italy (LE_0_: 83.4, 95% PI: 83.1-83.7), and Sweden (LE_0_: 82.9, 95% PI: 82.6-83.1).

For Canadian women during the same study period, LE_0_ rank in comparison to the same 11 healthiest nations fell to the 10^th^ place in 2008 from the peak rank of the 3^rd^ in 1985. Projections based on recent trends suggested that Canadian women would fall to the 11^th^ place by 2023 with an LE_0_ of 84.9 years (95% PI: 84.5-85.2). This predicted value was significantly lower than that of women in 8 peer countries including Japan (LE_0_: 90.1, 95% PI: 89.8-90.4), Italy (LE_0_: 88.1, 95% PI: 87.8-88.4), France (LE_0_: 87.9, 95% PI: 87.4-88.3), Spain (LE_0_: 87.8, 95% PI: 87.5-88.1), Switzerland (LE_0_: 87.1, 95% PI: 86.8-87.4), Australia (LE_0_: 87.0, 95% PI: 86.7-87.2), Austria (LE_0_: 86.7, 95% PI: 86.4-87.0), and New Zealand (LE_0_: 86.3, 95% PI: 86.1-86.5).

A closer examination of the slopes of the linear trends suggested that the downward slip in LE_0_ rankings for both Canadian men and women was a direct result of the relatively smaller annual gains (Table 2). Estimates based on 1985–2009 data period suggested that the LE_0_ gain of 85 days/year for Canadian men only outranked that of two other countries under comparison and was significantly smaller compared to that of seven of the 12 healthiest nations in the world, namely, New Zealand (123 days/year), Austria (110 days/year), Australia (108 days/year), Italy (107 days/year), Switzerland (105 days/year), France (100 days/year), and Norway (96 days/year). The annual gain of 48 days in LE_0_ for Canadian women during the study period of 1985–2009 ranked higher than only one country in the comparison set, and it was significantly slower in comparison to nine of the 12 healthiest countries, namely, Japan (90 days/year), New Zealand (88 days/year), Italy (87 days/year), Austria (86 days/year), France (79 days/year), Spain (78 days/year), Australia (77 days/year), Switzerland (65 days/year), and Norway (57 days/year).

**Table 2:** **Comparison of LE**_**0**_ **improvement trends by annual gain (1985–2009 for Canada, 1985–2010 for other OECD countries)** ^**§**^

	**Male**		**Female**	
**Rank**	**Country**	**Annual gain (days)**	**Country**	**Annual gain (days)**
**1**	New Zealand	123*	Japan	90*
**2**	Austria	110*	New Zealand	88*
**3**	Australia	108*	Italy	87*
**4**	Italy	107*	Austria	86*
**5**	Switzerland	105*	France	79*
**6**	France	100*	Spain	78*
**7**	Norway	96*	Australia	77*
**8**	Sweden	88	Switzerland	65*
**9**	Spain	86	Norway	57*
**10**	Canada	85	Sweden	54
**11**	Netherlands	82	Canada	48
**12**	Japan	70*	Netherlands	38

^**§**^Annual improvement of LE_0_ in days is derived from the regression slope.

*P-value from testing hypothesis of equal slopes in comparison to Canada is considered significant (*) if less than 0.0045 after Bonferroni correction.

**Figure 4:**
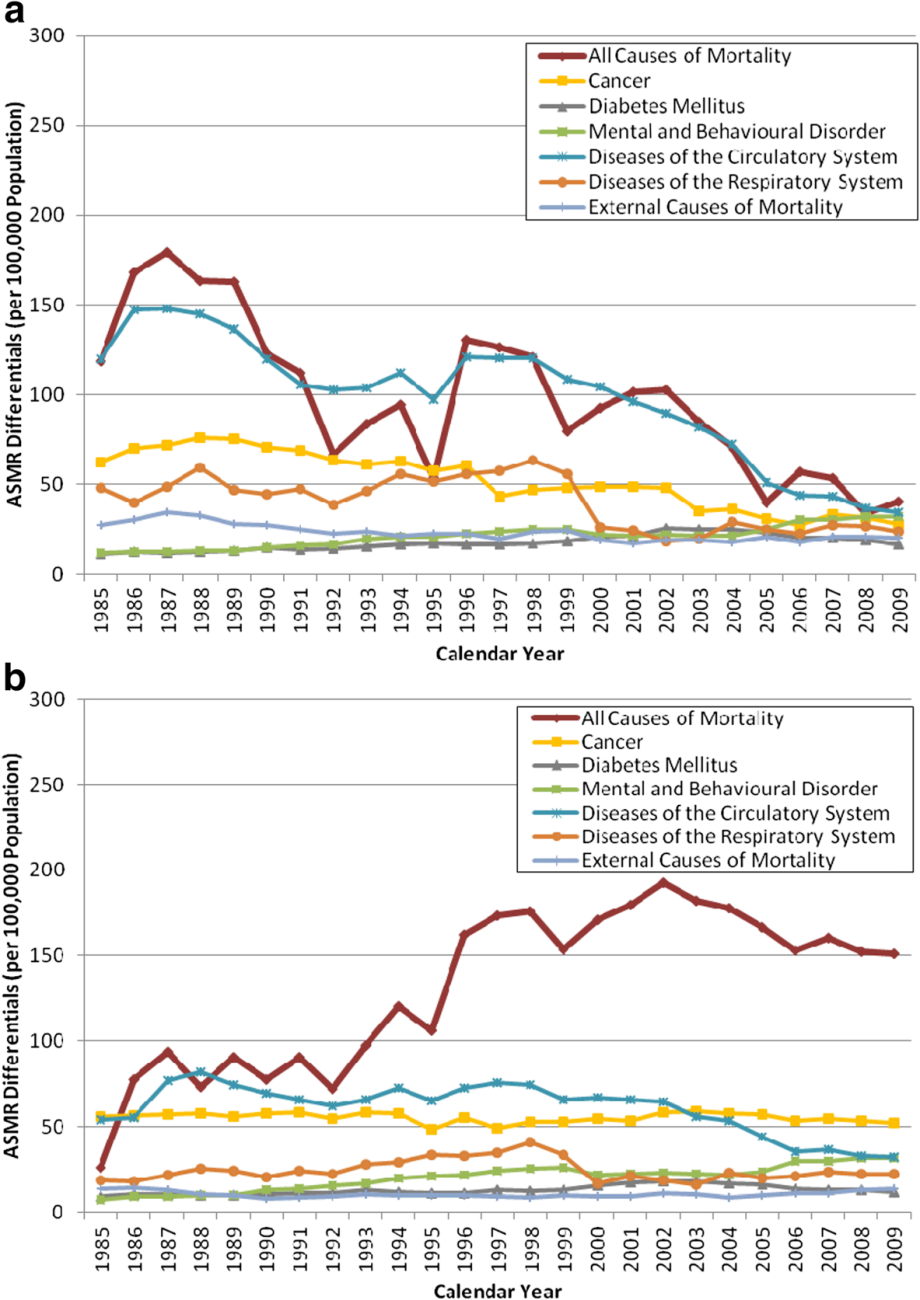
**Disadvantage of Canada Relative to the Best Performing Countries by Differences in Age-standardized Mortality Rate of Major Disease Categories, 1985-2009. a:** Disadvantage of Canada Relative to the Best Performing Countries by Differences in Age-standardized Mortality Rate of Major Disease Categories (Males), 1985–2009. **b:** Disadvantage of Canada Relative to the Best Performing Countries by Differences in Age-standardized Mortality Rate of Major Disease Categories (Females), 1985–2009.

The gap in annual cause-specific ASMR between Canada and the best performing country for men (Figure 4a) had been decreasing since the mid 1980’s for all causes of mortality and for most of the major disease categories examined. The narrowing of the gap in ASMR since 1985 for diseases of the circulatory system was particularly promising. One exception, however, was that Canadian men had been gradually fallen further behind those in countries with the lowest ASMR for mental and behavioural disorders since 1985. For females (Figure 4b), a widening of the gap between Canada and the best performing countries in terms of ASMR for all causes of mortality was observed and noticeable during the 1990’s. Similar to their male counterparts, a slight and gradual increase in the gap in mental and behavioural disorder related mortality risk in comparison to the best performing countries was seen.

**Figure 5:**
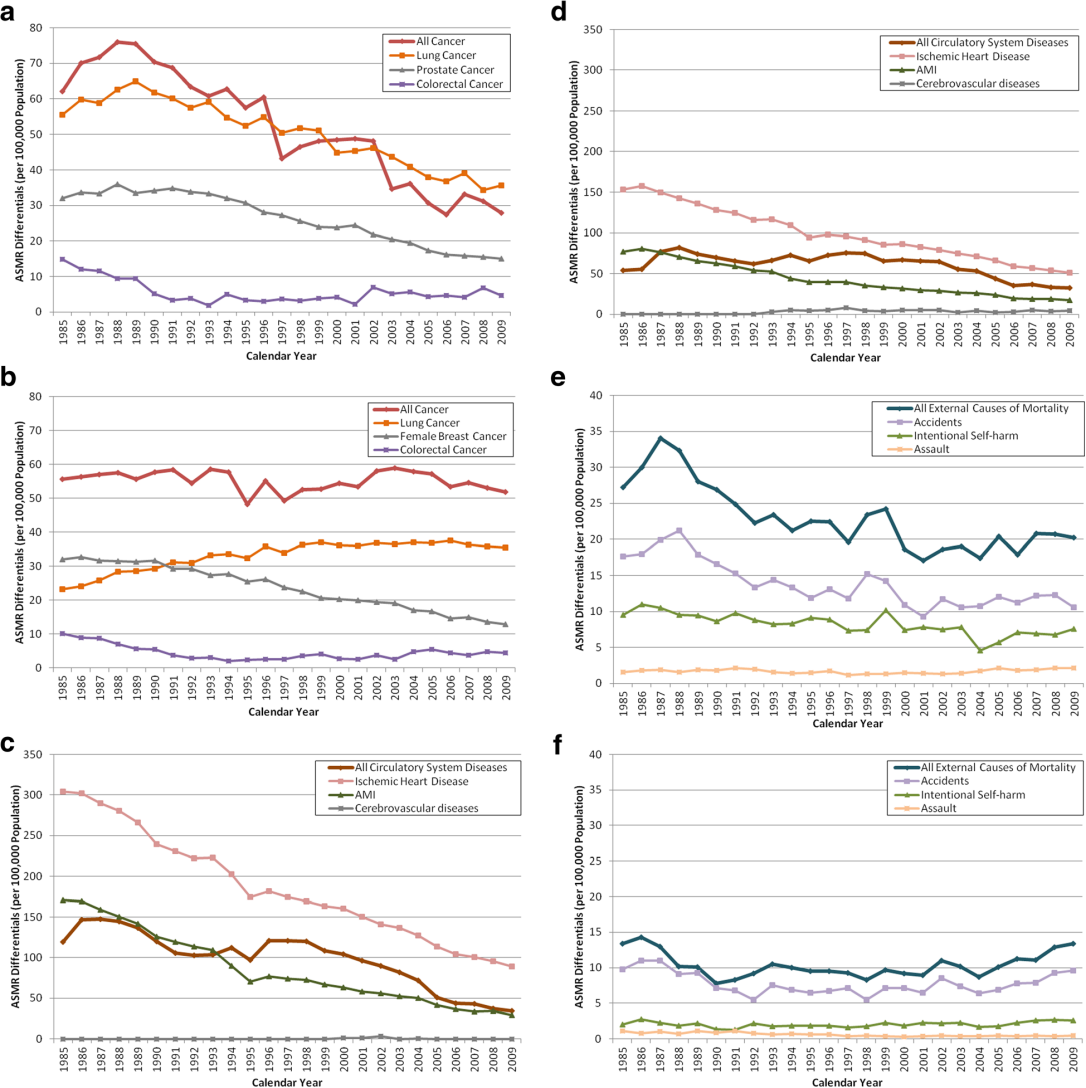
**Disadvantage of Canada relative to the best performing countries by differences in age-standardized mortality rate of selected disease conditions within major disease categories, 1985-2009. a:** Disadvantage of Canada Relative to the Best Performing Countries by Differences in Age-standardized Mortality Rate of Specific Cancer Types (Males), 1985–2009. **b:** Disadvantage of Canada Relative to the Best Performing Countries by Differences in Age-standardized Mortality Rate of Specific Cancer Types (Females), 1985–2009. **c:** Disadvantage of Canada Relative to the Best Performing Countries by Differences in Age-standardized Mortality Rate of Specific Circulatory System Diseases (Males), 1985–2009. **d:** Disadvantage of Canada Relative to the Best Performing Countries by Differences in Age-standardized Mortality Rate of Specific Circulatory System Diseases (Females), 1985–2009. **e:** Disadvantage of Canada Relative to the Best Performing Countries by Differences in Age-standardized Mortality Rate of Specific External Causes of Disease (Males), 1985–2009. **f:** Disadvantage of Canada Relative to the Best Performing Countries by Differences in Age-standardized Mortality Rate of Specific External Causes of Disease (Females), 1985–2009.

Looking at the gap in ASMR between Canada and the best performing countries for the specific disease conditions (Figure 5) within the major disease categories further revealed that between 1985 and 2009, Canadian women have fallen further behind their counterparts among the leading countries with respect to higher risks for lung cancer (Figure 5b) and all external causes of mortality, particularly accidents (Figure 5f).

**Figure 6:**
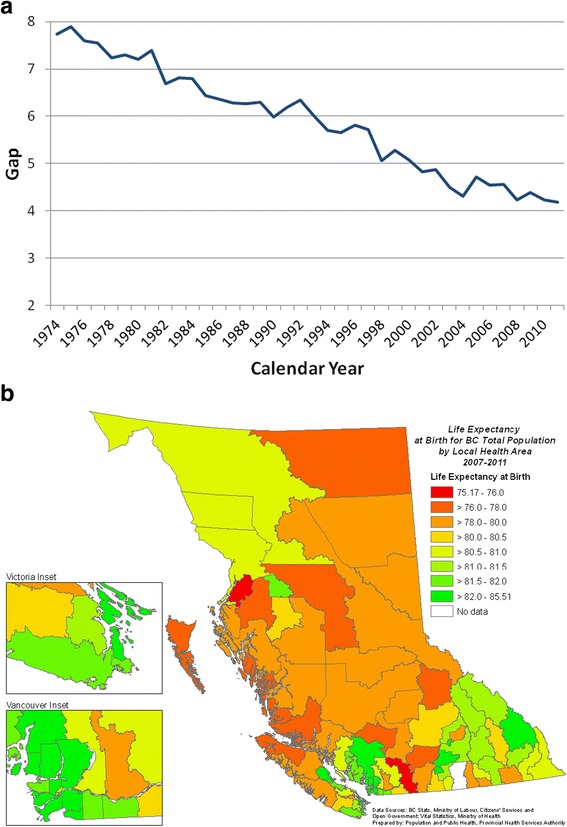
**Life expectancy in BC - sex gap (1975-2011) and geographical variations (2007-2011). a:** Gap in life expectancy at birth between BC females and males, 1975–2011. **b:** Life expectancy at birth for BC total population, by Local Health Area, 2007–2011.

Figure 6 showed the sex gap (Figure 6a) and geographical variations (Figure 6b) in LE_0_ in BC. Since reaching a peak of 7.8 years in 1975, the gap in life expectancy between females and males had been on a continuous decline. Geographically, local populations around the populous cities of Vancouver and Victoria and a few LHAs in the southeastern interior of the province (including Windermere, Armstrong-Spallumcheen, Central Okanagan, and Summerland) enjoyed higher longevity in 2007–2011, while those in the central and northern parts of the province had lower LE_0_.

SES is not evenly distributed in the province as demonstrated by Figure 1. Similar to the distribution of LE_0_, regions in and/or around the cities of Vancouver and Victoria (Lower Mainland and Southern Vancouver Island) as well as south-eastern interior regions have higher SES, whereas a large portion of the province’s central interior have low SES. Quantitative analysis confirmed that the average overall LE_0_ for LHAs in the lowest SES tertile of 78.6 years (95% CI: 78.0-79.3 years) was significantly lower than that for LHAs in the middle (LE_0_: 80.5 years, 95% CI: 79.8-81.1 years) and high (LE_0_: 82.2 years, 95% CI: 81.6-82.8 years) SES tertiles, with a 3.6-year gap between those in the extremity groups (Table 3). Similar associations between SES and LE_0_ as well as gaps in LE_0_ between the lowest and highest SES categories were observed for either sex (Table 3).

**Table 3:** **British Columbia regional average life expectancy at birth by regional socioeconomic status, 2007-2011**

**SES category**	**Total LE**_**0**_ **(95% CI)**	**Male LE**_**0**_ **(95% CI)**	**Female LE**_**0**_ **(95% CI)**
Low	78.6 (78.0 – 79.3)	76.6 (75.7-77.5)	81.1 (80.4-81.8)
Medium	80.5 (79.8 – 81.1)	78.2 (77.5-78.9)	82.8 (82.0-83.5)
High	82.2 (81.6 – 82.8)	80.2 (79.5-81.0)	84.2 (83.7-84.8)
LE_0_ Gap between low and high SES	3.6	3.6	3.1

SES: Socioeconomic status.

LE_0_: Life expectancy at birth.

CI: Confidence interval.

## Discussion

Canada’s position as one of the global leaders in LE_0_ has long been recognized [17,18] and was confirmed in this study. In 2008, Canadian men and women ranked 7^th^ and 8^th^, respectively among the 12 healthiest nations in the world. However, comparatively small annual gains in longevity contributed to the losing of their historical top 5 rankings, and the situation was anticipated to worsen into the next decade.

The current study’s finding of the downward slip in rankings for Canadian male and female populations among the healthiest populations in the world over time was consistent with that previously reported [18,19]. But looking ahead, despite the downward slip in ranks of Canadian men and women relative to their peers in other OECD countries, Canadian men and women will continue to live longer and reach a LE_0_ of 81.8 years (95% PI: 81.5-82.2) and 84.9 years (95% PI: 84.5-85.2) respectively by 2023, if current rates of increase continue.

Adding to the trend analysis, examination of cause-specific mortality rates suggested that the decline in LE_0_ ranking for Canadian women in comparison with their counterparts from the comparison OECD countries may partly be explained by the higher rates of mortality that Canadian women have been experiencing in recent years with cancers in the respiratory system (lung cancer in particular) and external causes, which included accidents such as those due to transport, falls, and poisoning, as well as intentional self-harm and assault. This finding is in agreement to national mortality data, which showed cancer and accidents were the 1^st^ and 5^th^ leading cause of death among Canadian women in 2011, respectively [20].

In Canada, the decline in cigarette smoking among females began about 20 years later than that among males [21]. This could in part explain the continued rise and yet to peak trend in lung cancer incidence among Canadian women, while the trend has reversed for Canadian men [22]. The rising incidence in lung cancer among Canadian women, in turn, could contribute to their higher rates of mortality due to this condition.

In addition, the interplay among a number of factors including employment status, environmental influences, socio-economic factors, ethnicity, and life-style may influence LE_0_ by affecting the prevalence of specific chronic conditions such as overweight and obesity and/or cause-specific mortality rates.

Geographic variations in LE_0_ were evident in BC, and the previously reported disparities in LE_0_ across socio-economic status (SES) despite the province’s highest rank in the country for this indicator [12] was evident. There was a significant gap of 3.6 years in LE_0_ for LHAs in the lowest compared to those in the highest SES tertile. A similar pattern was observed for males and females as well. In comparison, the gap found by using life expectancy data from 2006–2010 and the same SES categorization of the LHAs was 2.8 years (data not shown). This means that the life expectancy gap between the poor and the rich in the province showed signs of increase in recent years and that there is ample room for further improvement in longevity of British Columbians by addressing health disparity. Understanding the differences in health between population groups such as those reported here is critical to developing policies and programs that would reduce these differences. Surveillance of key population indicators such as LE_0_ also plays an on-going role of assessing any changes that may have resulted from the public health efforts.

Some limitations of data should be recognized in the interpretation of results from this study. LHA level SES index was used as a measure for LHA SES. As LHAs vary in geographic and population sizes as well as population characteristics, the overall SES index represented the average situation in each LHA. In the case of Vancouver city, one aggregate value of SES index was applied to all of its six LHAs with diverse population compositions and SES. Specifically, Downtown Eastside LHA hosts some of the poorest neighbourhoods in the province. Additionally, having to exclude some of the rural and remote LHAs in northern BC due to unavailability of SES data was expected to under-estimate the gap in SES across the province as they often have low SES. The interplay among these limitations was expected to attenuate the gap observed in LE_0_ by SES across LHAs overall, and it should be noted that any associations observed at the aggregate level might not necessarily hold true at the individual LHA level. The exclusion of a few additional LHAs due to lack of sex-specific LE_0_ data further attested to the diversity in population density across the different regions of the province. Sparsely populated regions in northern parts of BC were under-represented in the results and could attenuate sex-specific associations observed between LE_0_ and SES. The ecological nature of the analytical approach and the inability to control for other potential confounders also precluded any causal inferences made between mortality rates or social determinants of health and LE_0_ in either OECD-country comparisons or single jurisdiction analyses.

Linear regression was used in LE_0_ projections, and it was confirmed that the extent of the linear association of life expectancy with time as assessed by R^2^ of greater than 89% was high for all countries included in the analysis. This suggested that more than 89% of the variations in life expectancy could be explained by the regression models, and was in line with previous reports from different populations [23,24]. Although projections based on linear regression is sensitive to the timeframe of the yearly data points available, the use of data from a period that is twice as long as the projection period ensures reliable projection estimates if current trends continue.

## Conclusions

Canada continues to remain one of the selected OECD countries with longest living populations of males and females, despite the fact that the Canadian rank is sliding downwards among these countries. British Columbia, with the highest LE_0_ in the country, has an opportunity to address disparities in LE_0_ between the highest and lowest SES groups.

## Abbreviations

ASMR: Age-standardized mortality rates
BC: British Columbia
CI: Confidence interval
LE_0_: Life expectancy at birth
LHA: Local health area
OECD: Organization for Economic Cooperation and Development
PI: Prediction Interval
SES: Socioeconomic status
